# Support in acute situations when a community health nurse is called: experiences of older patients, their significant others, and involved healthcare professionals- a qualitative interview study

**DOI:** 10.1186/s12877-023-04331-0

**Published:** 2023-09-28

**Authors:** Elin-Sofie Forsgärde, Mattias Rööst, Anders Svensson, Bengt Fridlund, Carina Elmqvist

**Affiliations:** 1 Department of Health and Caring Sciences, Linnaeus University, PO Box 451, Växjö, 351 95 Sweden; 2 Center of Interprofessional Collaboration within Emergency Care (CICE), Linnaeus University, Växjö, 351 95 Sweden; 3 Ambulance Service, Region Kronoberg, PO Box 1207, Växjö, 352 54 Sweden; 4 Department for Research and Development, Region Kronoberg, PO Box 1223, Växjö, 351 12 Sweden; 5 Department of Clinical Sciences in Malmö, Family Medicine, Lund University, PO Box 50332, Malmö, 202 13 Sweden

**Keywords:** Aged, Ambulance service, Community health nurse, General practitioners, Nursing homes, Patients, Primary health care, Reflective lifeworld research, Support

## Abstract

**Background:**

Care decisions for older patients in acute situations are challenging to make, and there is limited knowledge of support in home healthcare settings, where older patients receive ongoing health care from, for example, community health nurses. Therefore, this study aimed to describe the support for all involved in acute situations when a community health nurse was called, as experienced by older patients, their significant others and healthcare professionals involved.

**Methods:**

The study was conducted using a phenomenological reflective lifeworld research approach, in which meanings of the study phenomenon were analyzed. The included participants were those who had been involved in acute situations. Twelve participants from four acute situations were interviewed. The participant included three older patients, one significant other, four community health nurses, one registered nurse student, one specialist in general practice, and two ambulance personnel, with one being a registered nurse and the other a specialist ambulance nurse.

**Results:**

Support in decision-making was received from the knowledge of temporality, which provided a comprehensive understanding based on past and present knowledge of the older patient. The knowledge of temporality allowed for the early detection of new symptoms and facilitated care decisions tailored to the older patient. There was a dependency on pre-existing mutual interpersonal support, and confidence developed through relational, caring, and medical competence.

**Conclusions:**

The advantages of temporality, confidence and mutual interpersonal support in acute situations highlight the importance of enhancing relational continuity in home healthcare settings and establishing a structural collaboration among community health nurses, specialists in general practice, and ambulance personnel. This collaboration aims to provide support for making decisions regarding tailored care.

## Introduction

Acute situations, such as deterioration in condition resulting from a newly occurring illness, injury or exacerbation of a chronic disease [1], increases with age due to age-related physiological changes [2]. Acute situations are not, per definition, associated with life-threatening conditions or the requirement for hospitalization, as long as appropriate care is received within a reasonable timeframe [3]. A global discussion has emphasized the necessity of developing health care tailored to an ageing population by extending the care provided in home healthcare settings [4, 5]. s. In this study, home healthcare settings refer to health care provided acutely or ongoing in patients’ ordinary homes, nursing homes, or primary healthcare (PHC) centers. In Sweden, patients who require assistance with daily living support and ongoing health care receive home health care in their ordinary homes until their needs surpass the capacity of home health care. At that point, they can apply for admission to a nursing home. Previous research has indicated that an increasing number of older patients calling for an ambulance ultimately receive care in home healthcare settings [6]. However, making care decisions is challenging [7], particularly for older patients, as age-related physiological changes and polypharmacy can result in non-specific symptoms [8] and risks of masking serious conditions [9]. Decisions that are not optimal for the older patient may result in harm and life-threatening conditions [10, 11]. Support during acute situations comprises, e.g. interpersonal support, guidelines, and checklists. However, there is currently a lack of joint decision-support in acute situations among, for example, community health nurses (CHNs) and ambulance personnel [11, 12]. CHNs refer to registered nurses (RNs) with and without various kinds of specialist education working in home healthcare settings.

Older patients have described experiencing acute situations with fear and lack of control, especially when they have cognitive and communication difficulties [13]. When making care decisions, older patients take an active or passive role depending on the availability of interpersonal support [14]. The absence of support in acute situations can generate feelings of abandonment [1]. Significant others, i.e. close friends or relatives of an older patient [15], experience acute situations similarly to older patients [1] but also differently because of their various roles [16]. In acute situations, significant others may feel unprepared and lack support in decision-making [17]. Significant others assume responsibility for communicating an older patient’s needs to healthcare professionals, i.e. CHNs, general practitioner (GP) specialists, and ambulance personnel, but at times they feel excluded from care decisions [1, 18].

Healthcare professionals have described feelings of uncertainty [7] and being under pressure in acute situations when assessing and deciding on the appropriate level-of-care for older patients [19, 20]. CHNs may receive collegial support during acute situations [21]. Additionally, in Sweden, a decision-support tool called the VISam checklist has been developed to support CHNs in level-of-care decisions in acute situations [12]. The VISam checklist is based on the triage system RETTS© [12], which is a five-level triage system developed for emergency departments (EDs) [22]. However, the VISam checklist differs from RETTS© by adapting the vital signs’ reference ranges to the conditions of older patients and utilizing two levels of severity (green and red). The green level suggests that older patients should remain at home or undergo an assessment by a GP specialist at a PHC center, while the red level suggests that the patient should be transported to an ED. The VISam checklist is regarded as supportive by CHNs as it facilitates structural assessment and supports care decisions [12]. However, knowledge of support from the VISam checklist is limited. Additionally, CHNs are often familiar with older patients and prefer them to remain at home for as long as possible [13, 23]. Moreover, CHNs occasionally experience a sense of disrespect from ambulance personnel when they decide to request an ambulance [23].

GP specialists have described being supported in decisions by weighing advantages and risks [24], and through receiving support from experienced CHNs who are familiar with the older patient [20]. It is described as challenging for GP specialists to make time for unscheduled visits and they therefore need to rely on the skill of CHNs in prioritizing and describing a situation. GP specialists further describe it as important to understand older patients’ wishes and reach a consensus during care decisions [20, 24]. Ambulance personnel have described that more complex decisions necessitate a greater level of support [25], including support from colleagues [26], and the use of checklists [27]. Ambulance personnel further describes that healthcare professionals at nursing homes occasionally have limited knowledge of the medical history of older patients, which complicates care decisions [28]. When a CHN’s decision appears insufficient, ambulance personnel sometimes question them [25] and also feel pressured to make decisions regarding transporting an older patient to an ED [28]. There is limited knowledge regarding the experiences of support in acute situations for all involved participants in home healthcare settings, where older patients receive ongoing home health care. Furthermore, the challenge of deciding on the appropriate level-of-care for older patients highlights the importance of further research.

## Aim

This study aims to describe support for all involved in acute situations when a CHN is called, as experienced by older patients, significant others, and healthcare professionals.

## Method

### Design and research approach

The study was conducted with a descriptive design using a reflective lifeworld research (RLR) approach. The RLR approach was used to describe meanings of the phenomenon: support in acute situations when a CHN is called. The RLR’s methodological principles were followed during the entire research process: being open and flexible to the phenomenon and bridling the understanding to maintain a reflective approach [29, 30].

## Setting

Sweden has a publicly funded healthcare system, and when someone needs home health care, an additional cost is charged based on the person’s income. Responsibility for health care in Sweden is divided between healthcare regions and municipalities [31].

The study was conducted in three municipalities in southern Sweden that implement the VISam checklist (Table 1).

**Table 1:** , An overview of the municipalities included in the study concerning sociodemographic characteristics and healthcare resources with information from Statistics Sweden [32]

	Municipality A	Municipality B	Municipality C
Area	1750 km²	890 km²	520 km²
Inhabitants	28 500	17 150	9 549
65 years or older	24%	20%	26%
Care facilities	Three primary healthcare centers One ambulance station One hospital	Two primary healthcare centers One ambulance station	Two primary healthcare centers One ambulance station

### Situations and participants

This study included participants from four different acute situations. These situations encompassed the possible care decisions suggested by the VISam checklist (Table 2). Acute situations arose when an older patient, a significant other or a healthcare professional identified that the older patient required immediate health care. The inclusion criteria were patients ≥ 65 years, who were receiving ongoing home health care or living in a nursing home, and who had experienced an acute illness, their significant others, and all healthcare professionals involved in the situations. The goal was to include all participants who were involved in the acute situations, encompassing a variety of acute situations, variations of symptoms among older patients, roles of healthcare professionals, and sizes of municipalities. The exclusion criteria were older patients unable to communicate without the support of a significant other during the interview. Contact persons, i.e. one CHN in each municipality (A–C), identified situations in which the inclusion criteria were met. The identified situations were discussed with the first author without revealing patient-sensitive information, which resulted in the inclusion of all identified situations.

Fifteen participants from the four situations were invited to participate in the study, of which twelve participants accepted: three patients all 80 years or older, one significant other, four CHNs, one student nurse, one GP specialist, and two ambulance personnel, i.e. one RN and one specialist ambulance nurse (SAN) (Table 2).

**Table 2:** An overview of the variations among and within the acute situations when a community health nurse was called, including municipalities and participants

Acute situation	1. An older patient received assessment and care by the community health nurse within the own home	2. An older patient received assessment and care by the community health nurse within a nursing home	3. An older patient received assessment by the community health nurse within the own home and was then transported by car to a specialist in general practice at the primary healthcare center	4. An older patient received assessment by the community health nurse and ambulance personnel within the own home and was then transported by an ambulance to the emergency department
Municipality	C	A	B	C
Patient	Male	Male	Female	Male- did not participate in the study
Symptom	Breathing difficulties, fever	Breathing difficulties, fever	Fatigue	Breathing difficulties, fever
Significant other				Wife
Community health nurse	Female	Female	Female	Female
Student nurse, 2nd semester				Female
Specialist in general practice at the primary healthcare center				Male
Registered nurse, in ambulance service				Female
Specialist ambulance nurse, in ambulance service				Male

### Data collection

The contact person then sent or read the study information letter to the older patients and significant others, who gave their consent to participate by sending a letter to the first author. After receiving the consent, the contact person and an ambulance service IT manager were informed and sent an information letter about the study to the healthcare professionals involved in the situation.

Individual interviews were performed between May and November 2018 and took place two to four weeks after the acute situation. The interviews were conducted in settings chosen by the participants, e.g. at their home or place of work, and were audio recorded. Prior to the interviews, a relationship was established. All interviews started with an open-ended question to describe the situations where the phenomenon, support in acute situations when a CHN is called, had occurred: “Please describe your experience of the actual situation”. During the interviews, the first author was open and flexible towards the phenomenon by being curious and asking follow-up questions in order to obtain in-depth information regarding lived experiences of the studied phenomenon, such as “Can you please tell me more?”. At the same time, the author bridled the own understanding to reduce the risk of jumping to conclusions. After the interviews, the first author transcribed the 30 − 90 min long interviews verbatim.

### Data analysis

The transcribed interviews in each situation, i.e. the situation description, were read multiple times in order to gain an overview of the phenomenon [33]. During the analysis, the first author was open and flexible towards the phenomenon by bridling the own understanding to maintain a reflective approach. An analysis was performed by going from parts of the material to the whole and back to the parts again in order to identify a new whole [29, 31]. Meaning units connected to the phenomenon were extracted, and the meanings within each unit were identified. Each meaning unit was understood in relation to the other meaning units, i.e. as a figure against its background. Similar meanings in each situation description were grouped into patterns. Patterns from all situation descriptions were then analyzed, and similarities and differences identified and grouped into separate clusters. Abstracted descriptions of each cluster were written down, and the phenomenon’s essence was identified by relating the cluster descriptions to each other in order to identify invariant meanings between them. Furthermore, the essence includes invariant meanings between older patients, their significant others, and healthcare professionals. Finally, the constituents, i.e. the contextual nuances, were identified within the essence [29]. The first and last authors held regular discussions during the analysis process to remain open to the studied phenomenon. The text was further reviewed by all authors, as well as at two seminars by researchers and doctoral students with experience of care and the RLR approach.

The results describe the phenomenon’s essence followed by the four constituents: (1) Knowledge of temporality as support in decisions, (2) Confidence as support in past and present relations, (3) Decision-support in the present situation, and (4) Dependency on mutual interpersonal support. Quotes are included in the constituents in order to contextualize the results [29].

## Results

### Essence

Support in acute situations when a CHN is called comprises temporality by integrating knowledge of the past and present, which is mediated in the encounter. The knowledge of temporality is unique in each situation. Through knowledge of temporality, past, and present symptoms are compared and intertwined to form a comprehensive understanding that supports decision-making. Comprehensive understanding is ensured of being transferred during care encounters in the near future. Confidence in relationships plays a supportive role in acute situations. Confidence is established through previous encounters characterized by competence and is further developed in the present encounter. In the present moment, support in actions and decisions is derived from past experience of acute situations and knowledge of appropriate courses of action. Conversely, support diminishes when there is a lack of knowledge and experience, and then a dependence on interpersonal support increases. Mutual interpersonal support is required in order to receive and give adapted care with multidimensional uniqueness in the present and near future, and it is dependent on the inclusion of all persons involved.

### Knowledge of temporality as support in decisions

Support in acute situations when a CHN is called entails the significance of temporality. Temporality involves comparing the knowledge of an older patient’s past with their present symptoms and supports decisions regarding tailored care. The past naturally carries over into the present through the experiences of older patients and their significant others, where the deterioration in their condition or the emergence of additional symptoms supports the idea that care is needed.

*”I was really… I didn´t eat I… was really exhausted…I’ve never lost so much weight… I needed help.”* Patient 1.

When deterioration in condition becomes chronic, it becomes a new norm that is used for comparison during future acute situations. The responsibility to call for an ambulance and handover knowledge of temporality, including past medical history and present symptoms to ambulance personnel, is transferred from older patients and their significant others to CHNs.

“*So I thought it was great that the nurse (CHN) came and said that we needed to call an ambulance… otherwise, I probably would not have sent him away (to the hospital)…”* Significant other 1.

Knowledge of an older patient’s past, including their medical history, living conditions, and support from significant others and healthcare professionals, is crucial for healthcare professionals to assess and makes decisions regarding tailored care. A comprehensive understanding is achieved through previous regular interactions with the older patient and their significant other, which allows for prompt recognition of any deterioration in their condition in the present.

*“This is someone (the older patient) I already know; I know what kind of diseases he has … I know whether his symptoms are new or whether he has had them before.”* CHN 2.

Comprehensive understanding of an older patient’s situation helps CHNs and GP specialists in expanding the foundation for decision-making, enhancing confidence, and providing support. At the same time, there is a risk that a gradual deterioration of an older patient’s condition may be perceived as normal and overlooked by those involved in the regular care. When knowledge of an older patient’s past is lacking, CHNs and GP specialists strive to develop a comprehensive understanding of the older patient’s situation, cognitive function, physical abilities, and social context before considering the medical information in the decision. In the present, when a decision about tailored care is unclear due to non-specific symptoms, the decision is facilitated by follow-up visits in the near future. These visits provide support and increase confidence in decisions by facilitating regular evaluation and adjustment of the care given.

*“We noticed that she was not alert…so we increased our visits to her.”* CHN 3.

When hospital care is required, CHNs transfer the knowledge of the older patient’s medical history and present symptoms to the ambulance personnel. The ambulance personnel are prepared to assess older patients without knowledge of their medical history. However, receiving information from the CHN provides valuable support during the transition of care. Information from the older patient, their significant others, and the CHNs assist the ambulance personnel in gaining a comprehensive understanding of the older patient, where knowledge from both the past and present supports decisions regarding tailored care.

*“They (CHN) know how it’s been before and knows the changes… it’s very important information.”* Ambulance personnel 1.

When the older patient returns home from the hospital, the CHN assesses their current symptoms in the follow-up encounter and asks for the significant other’s perspective about the care provided. This feedback serves to assess the effectiveness of past care decisions and gather insights that can support further decisions.

### Confidence as support in past and present relations

Support in acute situations when a CHN is called entails the establishment of confidence in past and present relations through relational, caring, and medical competence. Confidence in present relations is connected to the older patient’s perception of the healthcare professional’s capability to deliver tailored care. Support during acute situations is evident when older patients and significant others are familiar with and trust the CHNs and the GP specialist, knowing that help is available at any time. Established relationships additionally facilitate communication in the present.

*”Oh yes, he (the GP specialist) knows me… I think he’s good, like a normal human being…”* Patient 3.

Confidence serves as a form of support for the older patient and their significant other in establishing new relationships with healthcare professionals. This confidence stems from positive past experiences in similar situations and continues to grow during the encounter when the healthcare professional is perceived as competent. However, the perception of where competent care is obtained varies. Some individuals have increased confidence when receiving care at home, in a calm and familiar environment, while others feel more confident when receiving care at hospitals with healthcare professionals in close proximity and avoiding unfamiliar healthcare professionals in their own homes.

*“There are no disadvantages to being cared for at home… If they’re educated and know what they’re doing, I see no disadvantages with that.”* Patient 2.

Being familiar with the older patient and other healthcare professionals in acute situations provides support to the CHNs and the GP specialists as confidence is already established. Confidence in other healthcare professionals and their competencies supports communication and collaboration. Simultaneously, being familiar with other healthcare professionals also entails knowing their shortcomings and acting on them. Confidence in a healthcare professional’s descriptions of an older patient’s situation is based on their capacity to conduct trustworthy and reliable assessments, accurately describe the situation, and possess previous knowledge of the older patient. Upon receiving a description of an urgent situation, the older patient’s needs and the credibility of the information are evaluated in order to make decisions regarding tailored care.

*“I’ve worked here for so long that I also know the CHNs… I know how well they describe (patients’ situations) and their knowledge, whether or not it is deficient…”* GP specialist 1.

### Decision support in the present situation

Support in acute situations when a CHN is called means that support in decisions depends on experience, knowledge, and present symptoms. Knowing how to act when experiencing symptoms of acute illness, including knowing who to call and not hesitating to do so, provides older patients and their significant others with the necessary support in taking appropriate actions and decisions, despite being unaware of the underlying cause of the symptoms.

*“We have a safety alarm and also a telephone number to the CHN… they’re wonderful because they come and help us.”* Significant other 1.

Support in the present situation is available when the healthcare professionals possess prior experience and knowledge regarding the necessary actions in acute situations and when the older patient has specific symptoms. Specific symptoms serve as a common ground for healthcare professionals’ assessment of the care requirements for the older patient. In contrast, the absence of support arises when there is a lack of experience and knowledge or when the older patient presents non-specific symptoms. When there is a lack of experience and knowledge, partial support for actions and decisions can be derived from decision-support tools like the VISam checklist and triage. However, when older patients exhibit non-specific symptoms, the assistance provided by these decision-support tools is inadequate.

*“It’s good to have a tool to follow in stressful situations, so I can see whether I have checked everything, so I think it was good I could use the decision-support tool.”* Student nurse 1.

The CHNs seldom utilize the decision-support tools since they do not perceive them as supportive or do not find the need for additional support. Nevertheless, in instances where the decision-support tools are employed, there is a possibility that the CHNs and ambulance personnel may shift the responsibility for the decision onto the tool itself when doubts arise.

*“I would say my decisions are based on gut feelings (laughs), but it’s my experience, such as what you’ve seen and what you know….”* CHN 4.

The GP specialists obtain support through their experience and knowledge in conducting reasonable assessments and by their knowledge of the assignments of different level-of-care. In order to enhance support when older patients present non-specific symptoms, both the CHNs and the ambulance personnel seek interpersonal support. This is aimed at mitigating the chances of overlooking the severity of the older patient’s conditions and avoiding placing sole responsibility on themselves.

### Dependency on mutual interpersonal support

Support in acute situations when a CHN is called means dependency on mutual interpersonal support in the present and near future. Mutual interpersonal support means support that is both received and provided. The dependency on mutual interpersonal support arises when an older patient’s symptoms impact the ability to handle the situation autonomously, resulting in worry, fear, and existential contemplation. Mutual interpersonal support is necessary to address evolving needs in acute situations.

*“I was completely alone and had such a hard time feeling so weak… it felt better when a healthcare professional I knew arrived. It can be difficult when you’re alone….”* Patient 1.

On the one hand, the dependence on mutual interpersonal support involves frustration for the older patients and their significant others, as they find themselves in need of support from others. Furthermore, such support limits the opportunities for the older patients and their significant others to live in accordance with existing everyday routines, including a lack of trust for not receiving the promised care. On the other hand, significant others have no alternative but to offer support and provide care in the home during an acute situation.

*“When he’s at the hospital, I sleep… I do, yes I do.”* Significant other 1.

Mutual interpersonal support during acute situations is given and received by healthcare professionals from older patients, significant others, and other healthcare professionals. This support enhances the level of certainty when making decisions. There is a dependency on mutual interpersonal support in order to reach the goal of providing care for older patients in their homes, as they are unable to manage their own care independently. This support is given and received through conversations with older patients and their loved ones, where information is gathered and a consensus is reached regarding the required type of care. Decisions regarding tailored care are primarily based on the older patient’s subjective preferences and, secondarily, on objective symptoms.

*“There was nothing directly deviating, and he (the patient) didn’t want to go to the hospital, so we decided to wait until the morning for a GP specialist assessment.”* CHN 1.

When no consensus is reached and potential risks for continued care in the home are identified, decisions regarding hospital care are made. Mutual support from other healthcare professionals is provided and received through dialogues that involve multiple perspectives to support decisions. The support from other healthcare professionals alleviates the burden on CHNs and ambulance personnel, while also enhancing confidence in the decisions made.

*“They (CHNs) have made an assessment, and then we (ambulance personnel) make an assessment, and if we come to the same conclusion, it gives extra support to the decision.”* Ambulance personnel 2.

Support is received from the GP specialists by shouldering responsibility for the CHN’s decisions. When support from other healthcare professionals is lacking, the CHNs encounter challenges in determining appropriate care tailored to the needs of older patients.

## Discussion

Support in acute situations when a CHN is called includes knowledge of temporality, confidence in relations, the present situation, and mutual interpersonal support. Temporality as support integrates knowledge of the past and present situation into a comprehensive understanding that is transferred in the near future to other healthcare professionals. In the present situation, experiences and knowledge of acute situations as well as the older patient’s symptoms provide support in decision-making. The result contributes new knowledge that CHNs play a significant role in transferring the knowledge of temporality, owing to the value of this knowledge as a decision-support aspect in acute situations. Confidence in relations are supportive in acute situations and is established through relational, caring, and medical competence and further developed in the present encounter. Mutual interpersonal support combines the expertise of older patients, significant others, and healthcare professionals in order to tailor the care to an older patient in the present and the near future.

The following discussion focuses on ‘knowledge of temporality as support’ and ‘dependency on mutual interpersonal support’. The results show that having knowledge of temporality provides valuable support in acute situations for making decisions that align with an older patient’s needs. Temporality-based support is established through regular meetings, where a comprehensive understanding is developed, allowing for the early detection of new symptoms, and follow-up meetings in the near future. These results are in line with another study [34]. However, these results highlight the significant role of CHNs in transferring the knowledge of temporality, i.e. the comprehensive understanding of older patients to ambulance personnel. This comprehensive understanding encompasses information beyond what is documented in medical records, including an older patient’s preferences, goals, expectations, skills, and knowledge [35]. Knowledge of temporality is founded on continuity [36], which is a patient safety element that helps decrease preventable hospitalization [37] and mortality [38]. Relational continuity, which entails regular interactions with the same healthcare professionals, has been noted to promote tailored care that suits the specific needs of patients [35]. While relational continuity offers numerous advantages, there is a potential risk of overlooking gradual changes or maintaining care relationships where patients lack trust in a healthcare professional [39]. However, functional relational continuity strengthens the level of confidence between older patients and healthcare professionals, facilitating a comprehensive understanding of the patient’s circumstances [36]. In Sweden, only 35% of adult residents experience relational continuity with CHNs or GP specialists, whereas in other high-income countries, the percentage ranges from 80 to 95% [40]. The results highlight the benefits of temporality as a decision-support aspect. Unlike relational continuity, knowledge of temporality can be effectively transmitted to others. Temporality intertwines the past, present, and future into a cohesive entity [41]. It is an ever-present aspect of human consciousness, where the current situation is influenced by past experiences and anticipated future circumstances [42]. Accordingly, temporality brings a comprehensive understanding of an older patient, extending the grounds for decision-making and supporting care decisions. These results highlight the need to increase relational continuity in healthcare and leverage temporality as a means of support when making decisions in acute situations.

The results show a dependency on mutual interpersonal support during decision-making in acute situations, especially when older patients have non-specific symptoms and decision-support tools are insufficient. Moreover, this reliance encompasses both the capacity to receive and provide care. The necessity for mutual interpersonal support aligns with findings from other studies [7, 43]. Receiving and providing care depends on having confidence in others and their ability to take responsibility to support others, also referred to as *social support* [44]. Social support is categorized into emotional (caring, empathy, and trust), instrumental (concrete assistance), informational (information for solving problems), and appraisal support (constructive feedback) [45]. The results reveal that healthcare professionals provide emotional support to older patients and their significant others by actively listening to them. Additionally, they offer instrumental, informational, and appraisal support through activities such as assessment, communicating findings, and conducting follow-up visits. It is important to note that a lack of social support puts patients at risk, leading to a deficiency in emotional support, as well as potential instances of missed or delayed diagnoses due to inadequate instrumental, informational, and appraisal support [46, 47]. Older patients and their significant others have an important role in sharing their situations and goals with healthcare professionals to enable decisions regarding adapted care [48]. Care decisions are reached through a dynamic interaction between the desires and requirements of older patients and the caring and medical expertise of healthcare professionals [49]. The findings demonstrate a reliance on collaboration in acute situations, wherein healthcare professionals both provide and receive emotional, instrumental, and informational support from older patients, their significant others, and other healthcare professionals. Collaboration among healthcare professionals in acute scenarios involving older patients has been recognized as supportive in a previous study, as it combines profession-specific expertise with organizational benefits [7]. By combining the knowledge of CHNs regarding an older patient with ambulance personnel expertise regarding acute situations and the equipment and medication available [50], the decision-making processes of CHNs and ambulance personnel can be enhanced. Additionally, GP specialists and CHNs have the advantage of follow-up visits, allowing for regular adjustments and adaptations to care, which compensates for the absence of such opportunities for ambulance personnel [51]. Consequently, a structured collaboration among all parties involved in acute situations is crucial as a means of support, leveraging the expertise of older patients, their significant others, and healthcare professionals to their advantage.

### Methodological considerations

Methodological quality was achieved by using RLR’s methodological principles in the research process [29, 52]. The goal was to include variations of situations, participants, and municipalities to achieve an extensive description of the phenomenon. Possible limitations were, for example, limited variations in older patients’ symptoms and CHNs’ sex. However, more female than male CHNs were employed in home healthcare at the time of the study. Some of the situations were seldom conducted, and thus, when a situation was found that matched a predetermined situation, those involved were asked to participate regardless of, for example, the patient’s symptoms. On one hand, this could potentially influence the results and constrain the variations of components in the decisions made, given that three of the older patients exhibited similar symptoms. However, all care decisions for older patients are distinctive due to individual differences in the presentation and experience of symptoms, as well as the available support. The assessment is that these three situations were more dissimilar than alike, owing to the diversity in the residence of the elderly patients, the participants involved, and the varying severity of the symptoms. Moreover, since the study was phenomenon oriented, the overall variations of the phenomenon were rich.

The exclusion of one older was based on the assessment made by their significant other regarding the patient’s condition during the interview. This exclusion may have resulted in a potential lack of information within the study. However, this decision was carefully evaluated considering the potential risks of harm to the older patient. The request made by the significant other was honored, as she possessed knowledge about the older patient and her intention was to minimize any potential harm.

A strength of the study was that the interviews gave detailed and rich descriptions of the studied phenomenon. By using follow-up questions, the first author directed the participants to talk about the studied phenomenon. Furthermore, validity was achieved by analyzing meanings of the phenomenon and going back and forth from the parts to the whole multiple times to ensure that the results were well-grounded [52]. Objectivity was ensured by following the RLR’s methodological principles, in which the authors bridled their understanding in order to remain open, flexible, and curious about the phenomenon [52]. Recurrent discussions among the authors and during seminars were conducted in order to keep the phenomenon at a distance and allow understanding to evolve. Transferability of the results is possible due to the abstracted essence, which allows the reader to transfer the results into similar contexts and situations [52].

### Conclusions and implications

Support in acute situations when a CHN is called comprises knowledge of temporality, confidence, and mutual interpersonal support. A lack of support from existing decision-support tools is experienced when older patients have non-specific symptoms. Temporality as support includes a comprehensive understanding of an older patient, which enables early identification of new symptoms and facilitates the assessment of an older patient’s care needs in the present and near future. Confidence in relations provides support by relying on each other’s competence and decisions made. Finally, mutual interpersonal support combines the specific expertise of older patients, significant others, and healthcare professionals in order to tailor the care to an older patient’s unique needs.

Accordingly, several implications for increasing support in acute situations have been identified. The importance of temporality, confidence, and mutual interpersonal support as valuable forms of support in acute situations should be acknowledged. Active involvement of older patients and their significant others in mutual interpersonal support is crucial. Establishing a structured collaboration between CHNs, GP specialists, and ambulance personnel promotes informed care decisions by leveraging their respective expertise and organizational advantages. Lastly, there is a need to enhance relational continuity in home healthcare settings to facilitate the utilization of temporality as a form of support.

## Data Availability

The transcribed interviews are not publicly available due to not being considered in the ethical approval from the Swedish Ethical Review Authority. However, on reasonable request, the data are available from the corresponding author.

## List of abbreviations

CHN: Community health nurse.
ED: Emergency department.
GP: General practitioner.
PHC: Primary healthcare.
RLR: Reflective lifeworld research.
RN: Registered nurse.
SAN: Specialist ambulance nurse.
